# Supplementary material for: Targeting FAT1 Inhibits Carcinogenesis, Induces Oxidative Stress and Enhances Cisplatin Sensitivity through Deregulation of LRP5/WNT2/GSS Signaling Axis in Oral Squamous Cell Carcinoma
Source: Cancers (Basel). 2019 Nov 27;11(12):1883. doi: 10.3390/cancers11121883 (PMC6966489; doi:10.3390/cancers11121883)
Supplement: Supplementary file 1 [file cancers-11-01883-s001.zip › cancers-635624-supplementary-final/cancers-635624-Supplenmentary-final.docx]

Article

Targeting FAT1 Inhibits Carcinogenesis, Induces Oxidative Stress and Enhances Cisplatin Sensitivity through Deregulation of LRP5/WNT2/GSS Signaling Axis in Oral Squamous Cell Carcinoma

Tung-Nien Hsu ^1,2,†^, Chih-Ming Huang ^3,†^, Chin-Sheng Huang ^1,2^, Mao-Suan Huang ^1,2^, Chi-Tai Yeh ^4,5^, Tsu-Yi Chao ^4,6,7^, Oluwaseun Adebayo Bamodu ^4,5,^*


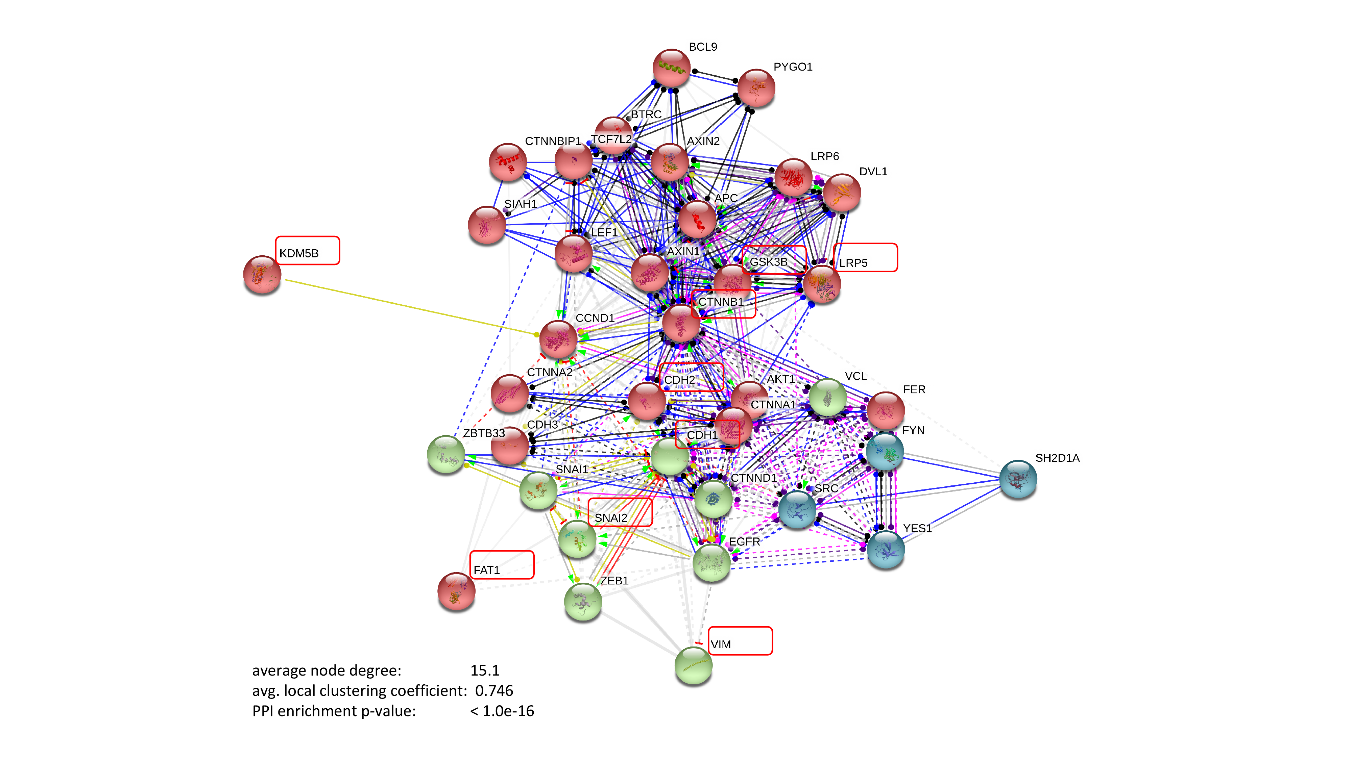


**Figure S1.** FAT1 by association is implicated in the oncogenicity and metastatic phenotype of OSCC cells. (**A**) Network visualization of molecular interaction between FAT1, CDH1, CDH2, CTNNB1, SNAI2, VIM, and KDM5B with an average node degree of 15.1^°^, average local clustering coefficient of 0.746, and protein-protein interaction (PPI) enrichment *p*-value of < 1.0 10^-16^. Red box, protein of interest.


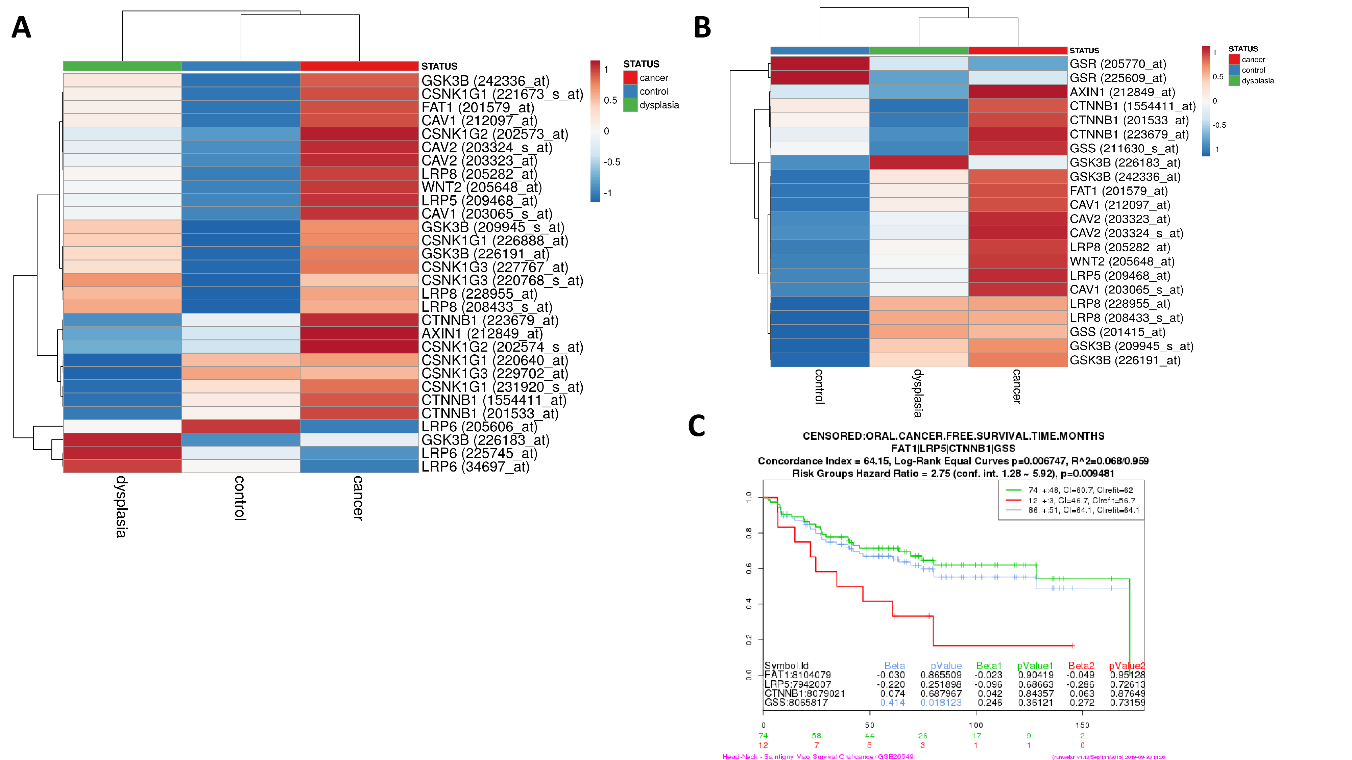


**Figure S2.** Enhanced FAT1 expression is associated with an activated LRP5 signalosome, dampened oxidative stress, and poor prognosis. Heatmaps of (**A**) FAT1, LRP5, LRP6, LRP8, WNT2, GSK3B, CTNNB1, CSNK1G1, CSNK1G2, CSNK1G3, AXIN1, CAV1, and CAV2, or (**B**) FAT1, LRP5, LRP8, GSK3B, CTNNB1, AXIN1, CAV1, CAV2, GSR, and GSS gene expression profile in OSCC cohort (*n* = 229 samples, 54675 genes) using the A-AFFY-44, AFFY_HG_U133_PLUS_2, E-GEOD-30784 dataset. Columns with similar annotations are collapsed by taking median inside each group. Rows are centered; unit variance scaling is applied to rows. Both rows and columns are clustered using correlation distance and average linkage. 30 rows, 3 columns. (**C**) Kaplan-Meier plot showing the association between the differential expression of FAT1/LRP5/CTNNB1/ GSS gene panel and overall survival of OSCC patients using the GSE26549 Head-Neck -Saintigny Mao Oral cancer cohort dataset (*n* = 86).


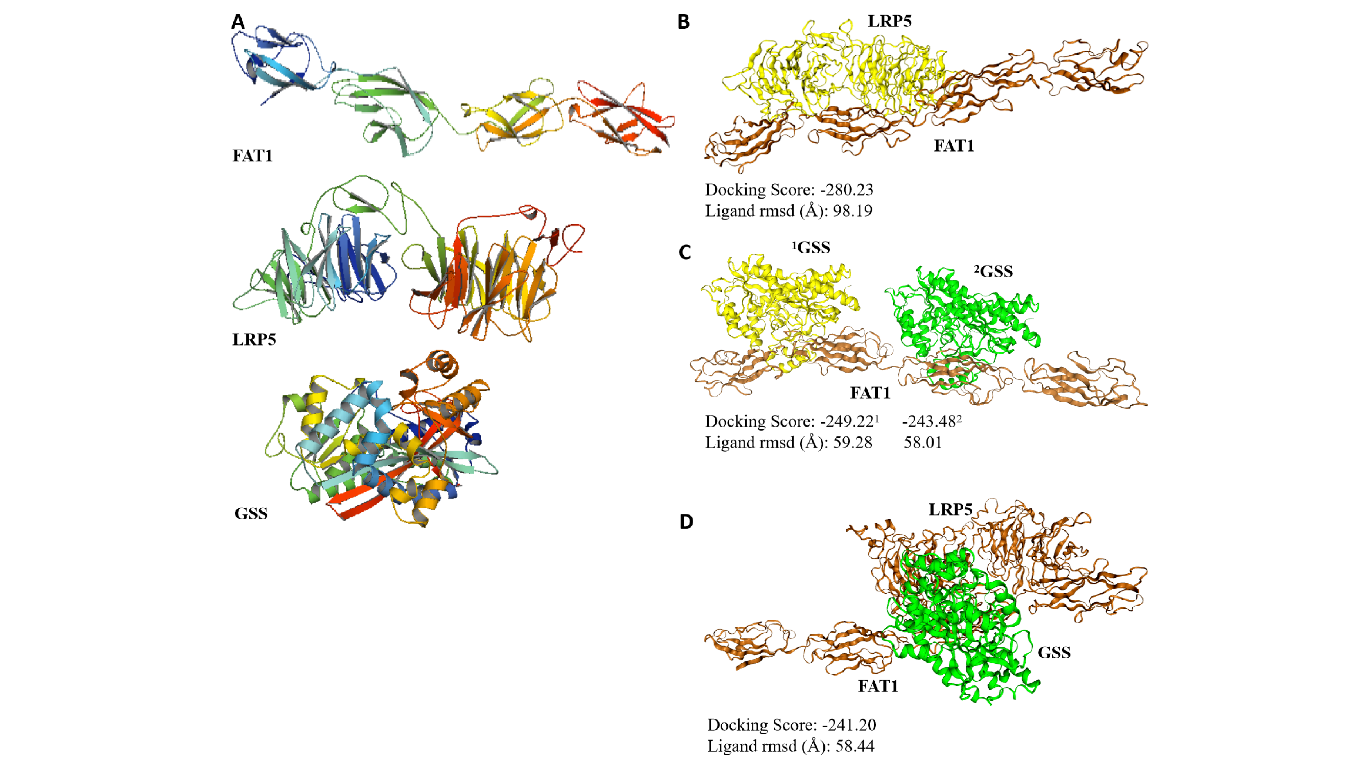


**Figure S3.** FAT1 interacts with LRP5 and GSS, to form FAT1/LRP5/GSS signalosome complex in OSCC cells. 3D visualization of the molecular structure of (**A**) FAT1, LRP5, and GSS, as well as direct protein-protein complex formation between (**B**) FAT1 and LRP5, (**C**) FAT1 and GSS, or (**D**) FAT1, LRP5, and GSS. Docking scores and ligand RSMDs are indicated. RMSD, root-mean-square deviation.


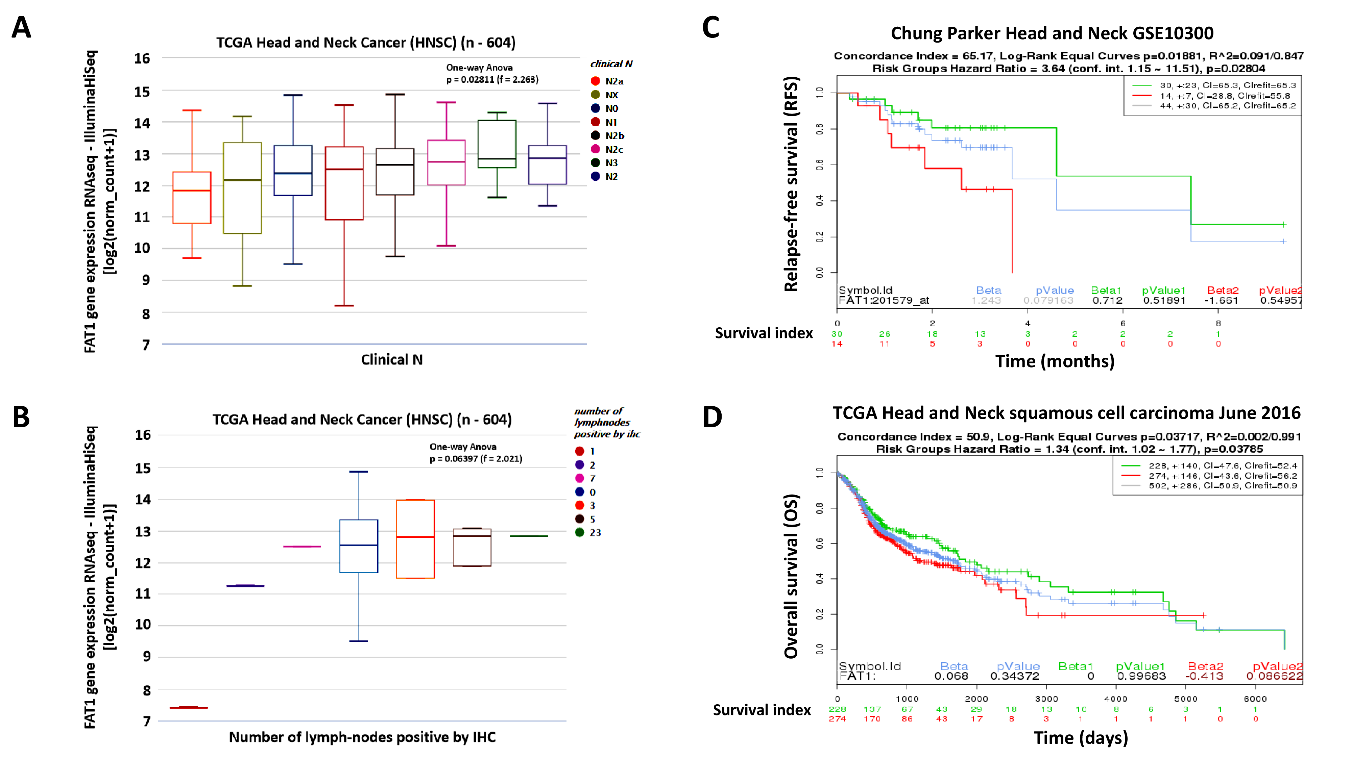


**Figure S4.** High FAT1 expression is positively correlated with nodal involvement and poor clinical outcome in patients with HNSC. Boxplot and whiskers plots of the correlation between (**A**) clinical node (N) stage, or (**B**) number of lymph-nodes positive by IHC, and the differential expression of FAT1 in TCGA-HNSC cohort. N, nodal; IHC, immunohistochemistry. Kaplan-Meier plots of the correlation between differential FAT1 expression and (**C**) relapse-free survival in the Chung Parker Head and Neck GSE10300 cohort, n = 44, or (**D**) overall survival in the TCGA-HNSC June 2016 cohort, n = 502.
