# Supplementary material for: Targeting FAT1 Inhibits Carcinogenesis, Induces Oxidative Stress and Enhances Cisplatin Sensitivity through Deregulation of LRP5/WNT2/GSS Signaling Axis in Oral Squamous Cell Carcinoma
Source: Cancers (Basel). 2019 Nov 27;11(12):1883. doi: 10.3390/cancers11121883 (PMC6966489; doi:10.3390/cancers11121883)

**Western Blot Raw Data**

**Targeting FAT1 inhibits carcinogenesis, induces oxidative stress and enhances cisplatin sensitivity through deregulation of LRP5/WNT2/GSS signaling axis in Oral Squamous Cell Carcinoma**

Tung-Nien Hsu, MD^1,2#^, Chih-Ming Huang, MD., PhD^3#^, Chin-Sheng Huang, MD^1,2^,

Mao-Suan Huang, MD^1,2^, Chi-Tai Yeh, PhD^4,5^, Tsu-Yi Chao, MD., PhD^4,6,7,^ Oluwaseun Adebayo Bamodu, MD., PhD^4,5*^

^1^ Division of Oral and Maxillofacial Surgery, Department of Dentistry, Taipei Medical

University - Shuang Ho Hospital, New Taipei City 235, Taiwan;

^2^ School of Dentistry, College of Oral Medicine, Taipei Medical University, Taipei City 110, Taiwan;

^3^Department of Otolaryngology, Taitung Mackay Memorial Hospital, Taitung City 950, Taiwan;

^4^Department of Hematology and Oncology, Cancer Center, Taipei Medical University - Shuang Ho Hospital, New Taipei City 235, Taiwan;

^5^Department of Medical Research & Education, Taipei Medical University – Shuang Ho Hospital, New Taipei City 235, Taiwan;

^6^Graduate Institute of Clinical Medicine, Taipei Medical University, Taipei City 110, Taiwan;

^7^Taipei Cancer Center, Taipei Medical University, Taipei City 110, Taiwan.

#Co-first authors.

* Corresponding: [16625@s.tmu.edu.tw](mailto:16625@s.tmu.edu.tw); Oluwaseun Adebayo Bamodu, MD., MS., PhD. Department of Hematology and Oncology, Cancer Center, Taipei Medical University -Shuang Ho Hospital, New Taipei City, Taiwan. Phone: +886-2-2490088 ext. 8742; FAX: 886-2-2248-0900;

**1.** Full-size blots of Figure 3D

**
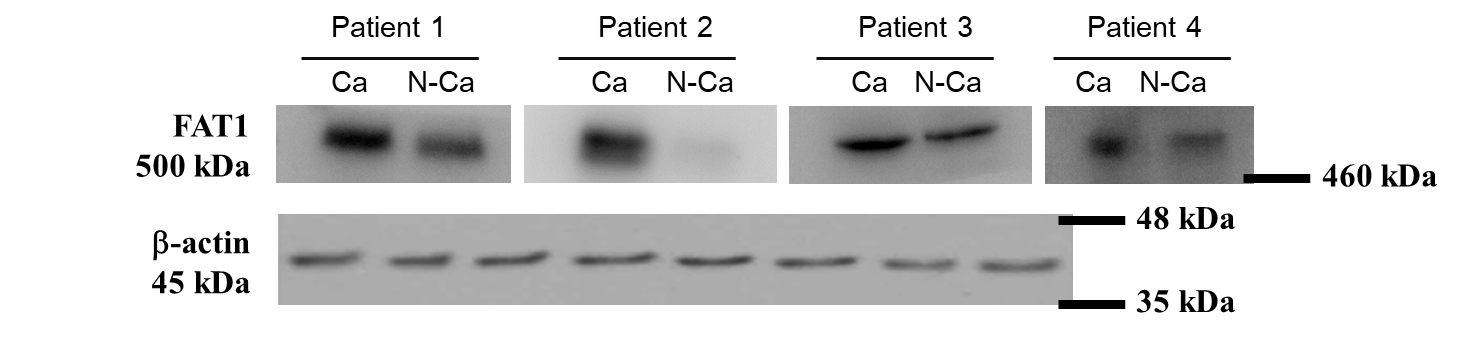
**

**2.** Full-size blots of Figure 4C

**
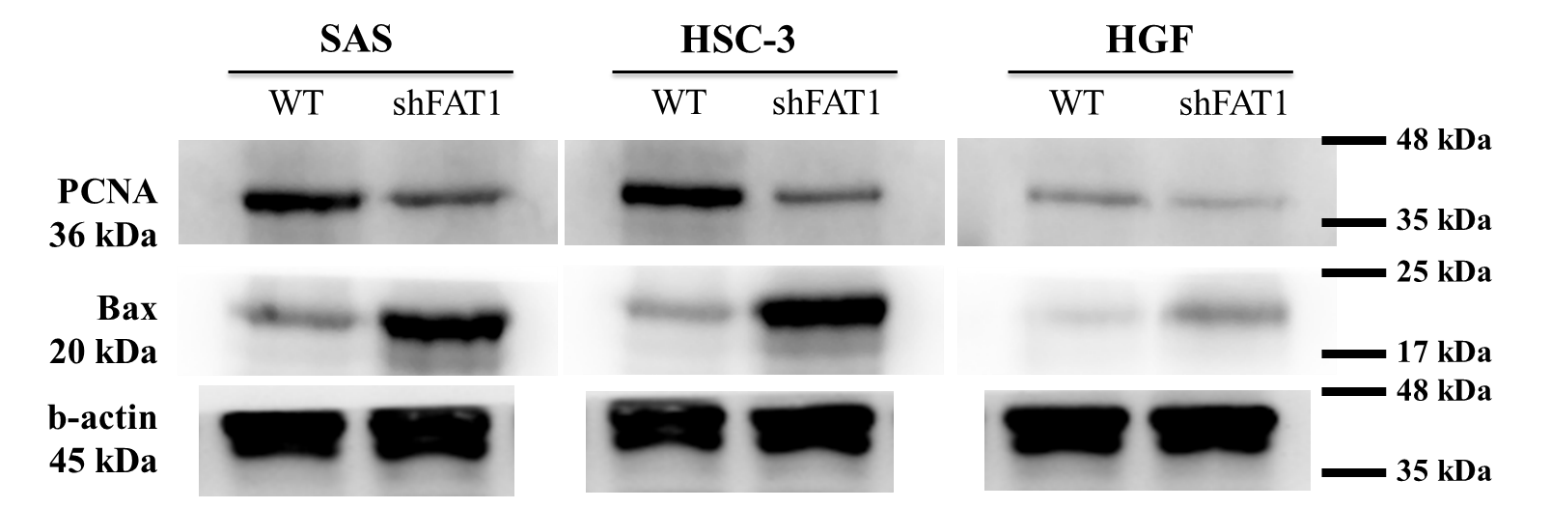
**

**3.** Full-size blots of Figure 6C, E and G


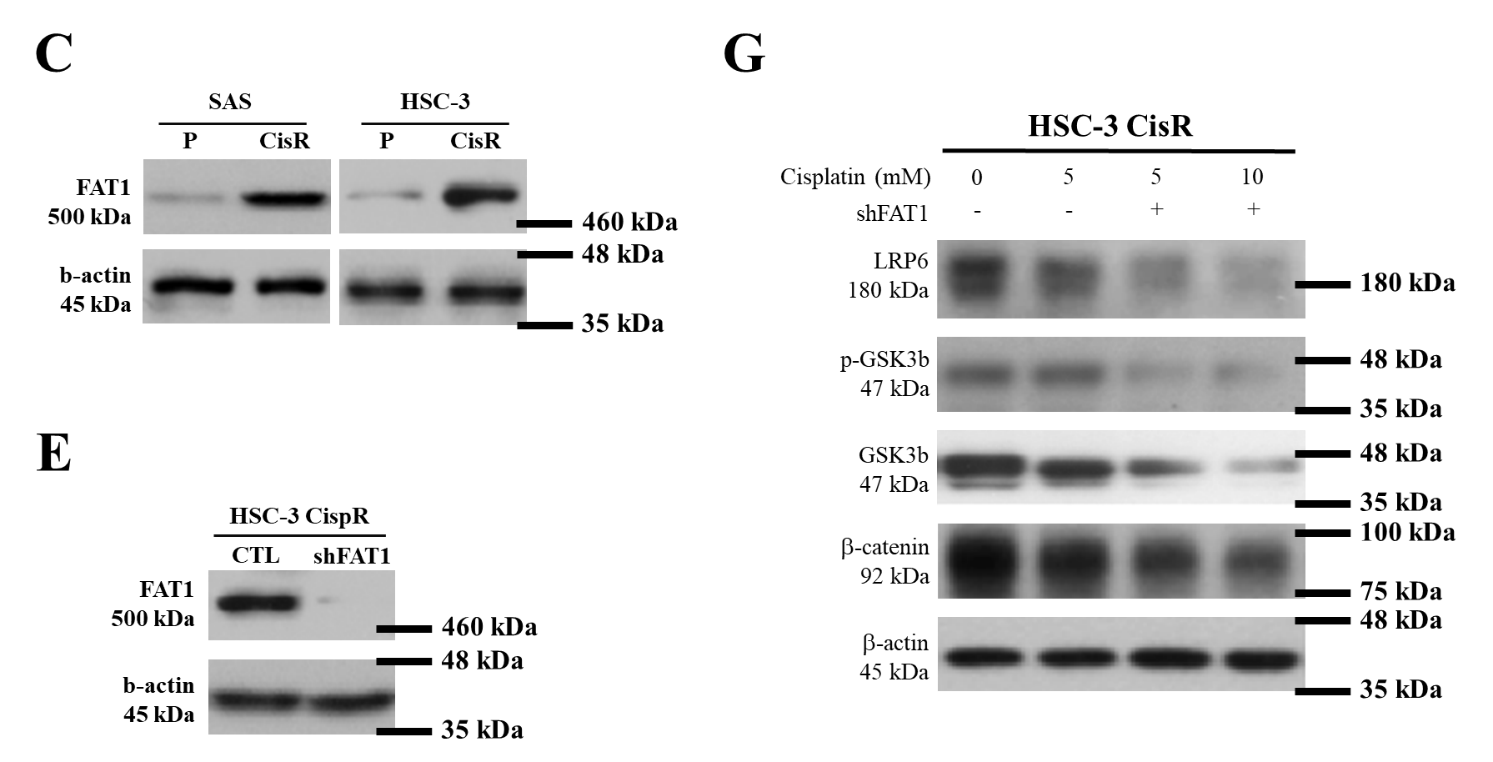

Supplement: Supplementary file 1 [file cancers-11-01883-s001.zip › cancers-635624-supplementary-final/cancers-635624-Western blot figures supplementarry.docx]
